# Outage Probability of MRC for *κ-μ* Shadowed Fading Channels under Co-Channel Interference

**DOI:** 10.1371/journal.pone.0166528

**Published:** 2016-11-16

**Authors:** Changfang Chen, Minglei Shu, Yinglong Wang, Ming Yang, Chongqing Zhang

**Affiliations:** 1 Shandong Computer Science Center (National Supercomputer Center in Jinan), Jinan 250014, China, Shandong Provincial Key Laboratory of Computer Networks, Jinan 250014, China; 2 College of Information Science and Engineering, Shandong University of Science and Technology, Qingdao 266590, China; West Virginia University, UNITED STATES

## Abstract

In this paper, exact closed-form expressions are derived for the outage probability (OP) of the maximal ratio combining (MRC) scheme in the *κ*-*μ* shadowed fading channels, in which both the independent and correlated shadowing components are considered. The scenario assumes the received desired signals are corrupted by the independent Rayleigh-faded co-channel interference (CCI) and background white Gaussian noise. To this end, first, the probability density function (PDF) of the *κ*-*μ* shadowed fading distribution is obtained in the form of a power series. Then the incomplete generalized moment-generating function (IG-MGF) of the received signal-to-interference-plus-noise ratio (SINR) is derived in the closed form. By using the IG-MGF results, closed-form expressions for the OP of MRC scheme are obtained over the *κ*-*μ* shadowed fading channels. Simulation results are included to validate the correctness of the analytical derivations. These new statistical results can be applied to the modeling and analysis of several wireless communication systems, such as body centric communications.

## Introduction

For wireless communication systems, diversity combining is an effective strategy to improve the performance with multipath fading and co-channel interference (CCI) [1]–[3]. When there is no CCI in a communication link, maximal ratio combining offers the best performance because it maximizes the SINR at the output of the combiner. When CCI is present, MRC is sub-optimal with respect to minimum mean squared error combining. However, in practice, MRC only requires the knowledge of the desired signal, and therefore it is easier to implement. For this reason, MRC is often applied in channels with CCI as it can achieve complexity and performance trade-off [4]–[7]. Moreover, correlations among the diversity branches will have a significant influence on the system performance [8]–[11].

The *κ*-*μ* fading distribution is proposed as a general multipath model which can represent the small-scale variation of a fading signal under line-of-sight (LOS) conditions [12]–[14]. This model includes some classical fading distributions as particular cases, such as one-sided Gaussian, Rayleigh, Rician and Nakagami-*m*. However, for many wireless communication systems, the LOS component is not deterministic, and its power randomly varies over time [15, 16]. This class of composite multipath and shadowing models are called LOS shadowed fading models, and an example of this model is the Rician shadowed fading model proposed in [17]. In the Rician shadowed fading model, it is assumed that the multipath fading follows the Rician distribution, and the shadowing experiences the Nakagami-*m* fading. Since the Rician distribution is a particular case of the *κ*-*μ* distribution, the *κ*-*μ* shadowed fading distribution can be derived by the same multipath and shadowing strategy used in the Rician shadowed fading models. In [18], it is assumed that the received scattered waves experience *κ*-*μ* fading, and the resultant dominant components are modeled by Nakagami-*m* distribution. This model provides a good experimental fit to body-centric communication channels where wireless devices are used in close proximity to the user’s body, the dominant or line-of-sight (LOS) components are susceptible to shadowing of the obstacles in the path trajectory. This situation may be further aggravated due to movements of the human body.

Outage probability is a common metric to measure the performance of wireless communication systems [1]. It is defined as the event that the SINR falls below a preset threshold. Much research has focused on the outage probability of MRC diversity reception with CCI [19]–[26]. Specifically, in [19], OP is derived, where both the desired signals and interfering signals experience Nakagami fading while simultaneously considering background noise. The peer-to-peer (P2P) share oriented routing schemes are proposed over multi-hop interference-constrained D2D networks [20], which maximize the average download rate over subscribers while controlling the interferences to the cellular user under a tolerable level. In [21], a closed form expression of OP for MRC is derived in *η* − *μ* fading channels with Rayleigh-faded interferers and background noise. Recently the closed form expressions of the OP are unveiled for *κ*-*μ* shadowed fading channels [22]–[24], either in interference-limited scenarios only, or without considering background noise [23, 24], and no results are available for *κ*-*μ* shadowed distribution with mixed background noise and interferers. For the classical distributions, the OP expressions are available [25, 26]. In [25], the OP results are derived under Nakagami-*m* fading channles in presence of both background noise and Rayleigh-faded interferers. In [26], the closed form expressions of OP are obtained in Nakagami-*q* fading channels with background white Gaussian noise and independent Rayleigh fading for interferers.

In this paper, we study the outage probability of the sum of the independent or correlated non-identically distributed squared *κ* − *μ* shadowed random variables in presence of CCI and the background noise. In [25, 26], it is revealed that the OP is related to the generalization of the moment generating function (MGF) of the fading distribution, i.e., the incomplete generalized MGF (IG-MGF). Therefore, it is of great interest to derive expressions of the IG-MGF for the MRC scheme over the *κ* − *μ* shadowed fading channels in this work. To this end, the probability density function of this sum is obtained in the form of a power series. Then the derived PDF is utilized for obtaining the IG-MGF of the received signal-to-interference-plus-noise ratio (SINR) of the MRC receiver. Based on the IG-MGF analysis, closed-form expressions for the OP of MRC can be derived with independent or correlated shadowed components in *κ* − *μ* shadowed fading channels. Finally, the statistical results are validated against the simulations.

The paper is organized as follows. In Section, the sum of independent or correlated squared *κ*-*μ* shadowed physical model is described, and the conditional PDF of SINR is derived. In Section, it is assumed that the dominant components of the *κ*-*μ* shadowed distribution are independent or correlated but arbitrarily distributed, and the unconditional PDF and the IG-MGF of the sum of the squared *κ*-*μ* shadowed distribution are obtained in closed form. In Section, the system model of *L*-antenna MRC receiver is presented, and based on the IG-MGF results, the outage probability is derived for MRC in *κ*-*μ* shadowed fading channels with co-channel interference and background noise, and the simulation results are included to validate the correctness of the analytical derivations in Section. Finally, we come to the conclusion of the paper in Section.

## Sum of *κ*-*μ* Shadowed Random Variables

The *κ*-*μ* shadowed fading distribution is a generalized physical model of the *κ*-*μ* distribution. For the *κ*-*μ* model, the dominant components of all the clusters are deterministic, but the dominant component of the *κ*-*μ* shadowed fading model randomly fluctuates within each cluster due to shadowing. In the *κ*-*μ* shadowed fading model, it is considered that the signal composes of clusters of multipath waves propagating in a nonhomogeneous manner. Within each cluster, the intracluster scattered waves have the random phases and similar temporal delays, but the intercluster delay-time spreads are relatively larger. Moreover, it is assumed identical power for the scattered waves, and arbitrary power for the dominant components.

For the *κ*-*μ* shadowed distribution, the signal power *W*_*l*_ can be denoted in terms of the in-phase and quadrature components as follows [22]

$$W_{l}=\sum_{i=1}^{\mu_{l}}\left[{\left(X_{i,l}+\xi_{l}p_{i,l}\right)}^{2}+{\left(Y_{i,l}+\xi_{l}q_{i,l}\right)}^{2}\right]$$ (1)

where *X*_*i*,*l*_ and *Y*_*i*,*l*_ are mutually independent Gaussian random processes with *E*[*X*_*i*,*l*_] = *E*[*Y*_*i*,*l*_] = 0 and $E\left[X_{i,l}^{2}\right]=E\left[Y_{i,l}^{2}\right]=\sigma^{2}$; *p*_*i*,*l*_ and *q*_*i*,*l*_ are real numbers. It is assumed all the dominant components are subject to the common random shadowing, represented by random variable *ξ*_*l*_, which is a Nakagami-*m* random variable with shaping parameter *m* and $E[\xi_{l}^{2}]=1$. For the deterministic LOS scenario, *ξ*_*l*_ = 1. For the physical model (1), each term of the sum represents one multipath cluster, and *μ*_*l*_ denotes the number of multipath clusters. *X*_*i*,*l*_ + *jY*_*i*,*l*_ is the circularly symmetric complex Gaussian random variable, which denotes the scattered component of the *i*th cluster with the total power 2*σ*^2^. *ξ*_*l*_ *p*_*i*,*l*_ + *jξ*_*l*_ *q*_*i*,*l*_ represents the dominant component of the *i*th cluster with power $p_{i,l}^{2}+q_{i,l}^{2}$.

From Eq (1), the expectation of *W*_*l*_ is $E\left[W_{l}\right]=\sum_{i=1}^{\mu_{l}}\left[p_{i,l}^{2}+q_{i,l}^{2}\right]+2\mu_{l}\sigma^{2}$. Conditioned on *ξ*_*l*_, *W*_*l*_ denotes the sum of 2*μ*_*l*_ independent noncentral chi-squared random variables, so the conditional PDF of *W*_*l*_ can be obtained as follows

$$f_{W_{l}|\xi_{l}}\left(w_{l}\right)=\frac{1}{2\sigma^{2}}{\left(\frac{w_{l}}{\xi_{l}^{2}d_{l}^{2}}\right)}^{\frac{\mu_{l}-1}{2}}\exp\left(-\frac{w_{l}+\xi_{l}^{2}d_{l}^{2}}{2\sigma^{2}}\right)I_{\mu_{l}-1}\left(\frac{\xi_{l}d_{l}}{\sigma^{2}}\sqrt{w_{l}}\right)$$ (2)

where $d_{l}^{2}=\sum_{i=1}^{\mu_{l}}\left[p_{i,l}^{2}+q_{i,l}^{2}\right]$, and *I*_*ν*_(⋅) is the modified Bessel function of the first kind. Define the following variable

$$\kappa_{l}≜\frac{d_{l}^{2}}{2\mu_{l}\sigma^{2}}$$ (3)

which denotes the ratio of the total power of the dominant components to the total power of the scattered waves. Furthermore, the instantaneous signal-to-interference-plus-noise (SINR) is defined as $\gamma_{l}={\bar{\gamma }}_{l}W_{l}/E\left[W_{l}\right]$ with the average SINR ${\bar{\gamma }}_{l}$. Following the standard procedure of transformation of variates, we obtain the conditional PDF of *γ*_*l*_

$$\begin{array}{ll} f_{\gamma_{l}|\xi_{l}}\left(\gamma_{l}\right) & =\frac{\mu_{l}{\left(1+\kappa_{l}\right)}^{\frac{\mu_{l}+1}{2}}}{{\bar{\gamma }}_{l}^{\frac{\mu_{l}+1}{2}}\kappa_{l}^{\frac{\mu_{l}-1}{2}}\xi_{l}^{\mu_{l}-1}}\gamma_{l}^{\frac{\mu_{l}-1}{2}}\exp\left(-\frac{\mu_{l}\left(1+\kappa_{l}\right)\gamma_{l}}{{\bar{\gamma }}_{l}}-\xi_{l}^{2}\mu_{l}\kappa_{l}\right)\\ & \cdot I_{\mu_{l}-1}\left(2\xi_{l}\mu_{l}\sqrt{\kappa_{l}\left(1+\kappa_{l}\right)}\sqrt{\frac{\gamma_{l}}{{\bar{\gamma }}_{l}}}\right) \end{array}$$ (4)

Consider the sum of *L* squared *κ*-*μ* shadowed variates

$$W≜\sum_{l=1}^{L}W_{l}$$ (5)

where *W*_*l*_, *l* = 1, …, *L* are independent or correlated non-identically distributed squared *κ*-*μ* shadowed random variables. In the diversity combining schemes, this sum is named MRC. Since *W* is a sum of $2\sum_{l=1}^{L}\mu_{l}$ non-central chi-squared variates, the conditional PDF of *W* can be derived as follows

$$\begin{array}{ll} f_{W|\xi_{1},\ldots ,\xi_{L}}\left(w\right) & =\frac{1}{2\sigma^{2}}{\left(\frac{w}{\sum_{l=1}^{L}\xi_{l}^{2}d_{l}^{2}}\right)}^{\frac{\sum_{l=1}^{L}\mu_{l}-1}{2}}\exp\left(-\frac{w+\sum_{l=1}^{L}\xi_{l}^{2}d_{l}^{2}}{2\sigma^{2}}\right)\\ & \cdot I_{\sum_{l=1}^{L}\mu_{l}-1}\left(\frac{\sqrt{\sum_{l=1}^{L}\xi_{l}^{2}d_{l}^{2}}}{\sigma^{2}}\sqrt{w}\right) \end{array}$$ (6)

Define the instantaneous SINR as $\gamma ≜\bar{\gamma }W/E\left[W\right]$ for the MRC scheme, where the average value of *W* is $E\left[W\right]=\sum_{l=1}^{L}d_{l}^{2}+2\sigma^{2}\sum_{l=1}^{L}\mu_{l}$, and $\bar{\gamma }$ denotes the average SINR. Using transformation of variates and Eq (3) for substitution, the conditional PDF of *γ* can be computed as follows

$$\begin{array}{ll} f_{\gamma |\xi_{1},\ldots ,\xi_{L}}\left(\gamma \right) & =\frac{{\left(\sum_{l=1}^{L}\mu_{l}\left(1+\kappa_{l}\right)\right)}^{\frac{\sum_{l=1}^{L}\mu_{l}+1}{2}}}{{\bar{\gamma }}^{\frac{\sum_{l=1}^{L}\mu_{l}+1}{2}}{\left(\sum_{l=1}^{L}\xi_{l}^{2}\kappa_{l}\mu_{l}\right)}^{\frac{\sum_{l=1}^{L}\mu_{l}-1}{2}}}\gamma^{\frac{\sum_{l=1}^{L}\mu_{l}-1}{2}}\\ & \cdot \exp\left(-\frac{\sum_{l=1}^{L}\mu_{l}\left(1+\kappa_{l}\right)\gamma }{\bar{\gamma }}-\sum_{l=1}^{L}\xi_{l}^{2}\kappa_{l}\mu_{l}\right)\\ & \cdot I_{\sum_{l=1}^{L}\mu_{l}-1}\left(2\sqrt{\sum_{l=1}^{L}\xi_{l}^{2}\kappa_{l}\mu_{l}\sum_{l=1}^{L}\mu_{l}\left(1+\kappa_{l}\right)}\sqrt{\frac{\gamma }{\bar{\gamma }}}\right) \end{array}$$ (7)

Defining ${\bar{\xi }}_{l}^{2}=\kappa_{l}\mu_{l}\xi_{l}^{2}$, the PDF can be easily obtained

$$f_{{\bar{\xi }}_{l}^{2}}\left(z_{l}\right)=\frac{z_{l}^{m-1}e^{-\frac{m}{\mu_{l}\kappa_{l}}z_{l}}}{\Gamma \left(m\right){\left(\frac{\mu_{l}\kappa_{l}}{m}\right)}^{m}}$$ (8)

Obviously, ${\bar{\xi }}_{l}^{2}$ follows Gamma distribution with the shape parameter *m*, and the scale parameter *μ*_*l*_*κ*_*l*_/*m*. Using this for substitution, it follows that

$$\begin{array}{ll} f_{\gamma |{\bar{\xi }}_{1},\ldots ,{\bar{\xi }}_{L}}\left(\gamma \right) & =\frac{{\left(\sum_{l=1}^{L}\mu_{l}\left(1+\kappa_{l}\right)\right)}^{\frac{\sum_{l=1}^{L}\mu_{l}+1}{2}}}{{\bar{\gamma }}^{\frac{\sum_{l=1}^{L}\mu_{l}+1}{2}}{\left(\sum_{l=1}^{L}{\bar{\xi }}_{l}^{2}\right)}^{\frac{\sum_{l=1}^{L}\mu_{l}-1}{2}}}\gamma^{\frac{\sum_{l=1}^{L}\mu_{l}-1}{2}}\\ & \cdot \exp\left(-\frac{\sum_{l=1}^{L}\mu_{l}\left(1+\kappa_{l}\right)\gamma }{\bar{\gamma }}-\sum_{l=1}^{L}{\bar{\xi }}_{l}^{2}\right)\\ & \cdot I_{\sum_{l=1}^{L}\mu_{l}-1}\left(2\sqrt{\sum_{l=1}^{L}{\bar{\xi }}_{l}^{2}\sum_{l=1}^{L}\mu_{l}\left(1+\kappa_{l}\right)}\sqrt{\frac{\gamma }{\bar{\gamma }}}\right) \end{array}$$ (9)

## Incomplete Generalized MGF of the *κ*-*μ* Shadowed Variates

### IG-MGF of the independent non-identically distributed *κ*-*μ* shadowed variates

**Definition 1** *Incomplete generalized moment-generating function (IG-MGF): Consider a continuous random variable X with PDF* *f*_*X*_(⋅) *and CDF* *F*_*X*_(⋅). *The IG-MGF of X*, *if it exists*, *is defined as* [27]

$$G\left(a,b;\epsilon \right)=\int_{\epsilon }^{\infty }x^{a}e^{bx}f_{X}\left(x\right)dx$$ (10)

*where* *b* ∈ **C**, *ϵ* ∈ **R**, *ϵ* ≥ 0, *and a is a nonnegative integer*.

From Eq (10), it can be seen that several important statistical functions of *X* can be included in Definition 1. For example, the moment generating function can be expressed as *G*(0, *b*; 0), and the complementary CDF can be derived by *G*(0, 0; *ϵ*). The generalized and marginal MGF can be obtained by *G*(*a*, *b*; 0) and *G*(0, *b*; *ϵ*), respectively.

**Definition 2** *Complementary IG-MGF: The complementary IG-MGF* $\tilde{G}\left(a,b;\epsilon \right)$ *of a continuous random variable* *X* *is defined as follows* [27]

$$\tilde{G}\left(a,b;\epsilon \right)=\int_{0}^{\epsilon }x^{a}e^{bx}f_{X}\left(x\right)dx$$ (11)

In view of Definition 2, as a particular case, the CDF *F*_*X*_(*x*) of *X* can be given by $\tilde{G}\left(0,0;x\right)$.

For an *L*-branch MRC scheme, the received instantaneous SINR of the receiver is given by $\gamma =\sum_{l=1}^{L}\gamma_{l}$, where it is assumed that each branch experiences *κ*-*μ* shadowed fading with an instantaneous SINR *γ*_*l*_, *l* = 1, …, *L*. The moment generating function of the received SINR *γ* is expressed as

$$M_{\gamma }\left(s\right)=E_{\gamma }\left[e^{s\gamma }\right]$$ (12)

First, it is assumed that the dominant components of the *κ*-*μ* shadowed random variables, i.e., ${\bar{\xi }}_{l}^{2}$ are mutually independent, and the PDF of $Z=\sum_{l=1}^{L}{\bar{\xi }}_{l}^{2}$ can be given by [28]

$$f_{Z}\left(z\right)=\prod_{l=1}^{L}{\left(\frac{m\beta_{0}}{\mu_{l}\kappa_{l}}\right)}^{m}\sum_{k=0}^{\infty }\frac{\delta_{k}z^{mL+k-1}e^{-z/\beta_{0}}}{\beta_{0}^{mL+k}\Gamma \left(mL+k\right)}$$ (13)

where *β*_0_ = min{*μ*_*l*_*κ*_*l*_/*m*}, Γ(⋅) is the Gamma function, and *δ*_*k*_ can be derived by the following formula

$$\delta_{k+1}=\frac{m}{k+1}\sum_{i=1}^{k+1}\left[\sum_{j=1}^{L}{\left(1-\frac{m\beta_{0}}{\mu_{j}\kappa_{j}}\right)}^{i}\right]\delta_{k+1-i},\;k=0,1,2,\ldots$$ (14)

where *δ*_0_ = 1. Define $\zeta =\sum_{l=1}^{L}\mu_{l}$, $\eta =\sum_{l=1}^{L}\mu_{l}\left(1+\kappa_{l}\right)$, then the conditional PDF of *γ* in Eq (9) can be expressed as

$$f_{\gamma |Z}\left(\gamma \right)=\frac{\eta^{\frac{\zeta +1}{2}}}{{\bar{\gamma }}^{\frac{\zeta +1}{2}}e^{\frac{\eta }{\bar{\gamma }}\gamma }}z^{-\frac{\zeta -1}{2}}\gamma^{\frac{\zeta -1}{2}}e^{-z}\cdot I_{\zeta -1}\left(2\sqrt{\frac{\eta \gamma }{\bar{\gamma }}}\sqrt{z}\right)$$ (15)

Averaged upon *Z*, we obtain the unconditional PDF of *γ*

$$\begin{array}{ll} f_{\gamma }(\gamma ) & =\overset{\infty }{\underset{0}{\int }}f_{\gamma |Z}(\gamma )f_{z}(z)dz\\ & =C{\left(\frac{\eta }{\bar{\gamma }}\right)}^{\frac{\zeta +1}{2}}\gamma^{\frac{\zeta -1}{2}}e^{-\frac{\eta }{\bar{\gamma }}\gamma }\sum_{k=0}^{\infty }D_{k}\Theta (\gamma )dz \end{array}$$ (16)

where $C=\prod_{l=1}^{L}{\left(m\beta_{0}/\mu_{l}\kappa_{l}\right)}^{m}$, $D_{k}=\delta_{k}/\beta_{0}^{mL+k}\Gamma \left(mL+k\right)$, and

$$\Theta \left(\gamma \right)=\int_{0}^{\infty }z^{mL+k-\frac{\zeta }{2}-\frac{1}{2}}e^{-\frac{1+\beta_{0}}{\beta_{0}}z}I_{\zeta -1}\left(2\sqrt{\frac{\eta \gamma }{\bar{\gamma }}}\sqrt{z}\right)dz$$ (17)

By using [29], the integral in Eq (17) can be solved, and substituting it into Eq (16) yields

$$f_{\gamma }\left(\gamma \right)=C{\left(\frac{\eta }{\bar{\gamma }}\right)}^{\zeta }\gamma^{\zeta -1}e^{-\frac{\eta }{\bar{\gamma }}\gamma }\sum_{k=0}^{\infty }{\tilde{D}}_{k}\cdot {}_{1}F_{1}\left(mL+k;\zeta ;\frac{\eta \beta_{0}\gamma }{\left(1+\beta_{0}\right)\bar{\gamma }}\right)$$ (18)

where ${\tilde{D}}_{k}=\delta_{k}/{\left(1+\beta_{0}\right)}^{mL+k}\Gamma \left(\zeta \right)$, and _1_*F*_1_(*a*; *b*; *z*) is the confluent Hypergeometric function [30]. From Eqs (12) and (18), the moment generating function of *γ* is given by

$$M_{\gamma }\left(s\right)=C{\left(\frac{\eta }{\bar{\gamma }}\right)}^{\zeta }\sum_{k=0}^{\infty }{\tilde{D}}_{k}\int_{0}^{\infty }\gamma^{\zeta -1}e^{-\left(\frac{\eta }{\bar{\gamma }}-s\right)\gamma }\cdot {}_{1}F_{1}\left(mL+k;\zeta ;\frac{\eta \beta_{0}\gamma }{\left(1+\beta_{0}\right)\bar{\gamma }}\right)d\gamma$$ (19)

Using the properties of the linearity and frequency shifting of the Laplace transform, it follows that

$$\begin{array}{ll} M_{\gamma }(s) & =ℒ[f_{\gamma }(\gamma );-s]\\ & =C{\left(\frac{\eta }{\bar{\gamma }}\right)}^{\zeta }\sum_{k=0}^{\infty }{\tilde{D}}_{k}ℒ\left[\gamma^{\zeta -1}{}_{1}F_{{}_{1}}\left(mL+k;\zeta ;\frac{\eta \beta_{0}\gamma }{(1+\beta_{0})\bar{\gamma }}\right);\frac{\eta }{\bar{\gamma }}-s\right] \end{array}$$ (20)

The Laplace transform in Eq (20) can be identified with [31], so the moment generating function of *γ* can be expressed as

$$M_{\gamma }\left(s\right)=C{\left(\frac{\eta }{\bar{\gamma }}\right)}^{\zeta }\sum_{k=0}^{\infty }{\bar{D}}_{k}{\left(\frac{\eta }{\bar{\gamma }}-s\right)}^{mL+k-\zeta }{\left(\frac{\eta }{\bar{\gamma }}-\frac{\eta \beta_{0}}{\left(1+\beta_{0}\right)\bar{\gamma }}-s\right)}^{-(mL+k)}$$ (21)

where ${\bar{D}}_{k}=\delta_{k}/{\left(1+\beta_{0}\right)}^{mL+k}$. For the diversity order and coding gain calculation, following the similar steps in [15], we can obtain the asymptotic bit error rate (BER) for *M*-ary quadrature amplitude modulation as follows

$$P_{e}\left(\bar{\gamma }\right)=\frac{2^{\zeta +1}C\left(1-\frac{1}{\sqrt{M}}\right)\Gamma \left(\zeta +\frac{1}{2}\right)}{\log_{2}\left(M\right)\sqrt{\pi }\zeta a_{1}^{2\zeta }}\sum_{k=0}^{\infty }{\tilde{D}}_{k}{\left(\frac{\eta }{\bar{\gamma }}\right)}^{\zeta }$$ (22)

At high *SINR*, the average BER of the communication system can be expressed by $P_{e}\left(\bar{\gamma }\right)={\left(G_{c}\bar{\gamma }\right)}^{-G_{d}}$, where *G*_*c*_ and *G*_*d*_ denote the coding gain and diversity order. Thus, it follows that

$$G_{d}=\zeta ,G_{c}={\left[\frac{\log_{2}\left(M\right)\sqrt{\pi }\zeta a_{1}^{2\zeta }}{2^{\zeta +1}\eta^{\zeta }C\left(1-\frac{1}{\sqrt{M}}\right)\Gamma \left(\zeta +\frac{1}{2}\right)\sum_{k=0}^{\infty }{\tilde{D}}_{k}}\right]}^{1/\zeta }$$ (23)

In view of the fact that $G\left(a,b;0\right)=G\left(a,b;\epsilon \right)+\tilde{G}\left(a,b;\epsilon \right)$, we can obtain G(a,b;ϵ) once G(a,b;0) and $\tilde{G}\left(a,b;\epsilon \right)$ are derived.

**Lemma 1** *Consider the sum of L independent non-identically distributed squared* *κ*-*μ* *shadowed variates with the instantaneous SINR* *γ*. *For* *a* ≥ 0, *Re*(*b*) < 0, *the generalized MGF of* *γ*, *i.e.*, $G_{\gamma }\left(a,b;0\right)$, *can be expressed as*

$$\begin{array}{ll} G_{\gamma }\left(a,b;0\right) & ={\left(-1\right)}^{\zeta }C{\left(\frac{\eta }{\bar{\gamma }}\right)}^{\zeta }\sum_{k=0}^{\infty }{\bar{D}}_{k}\sum_{i=0}^{a}\frac{a!{\left(c_{1}+k-i+1\right)}_{i}\;{\left(c_{2}-k-a+i+1\right)}_{a-i}}{i!(a-i)!}\\ & \cdot {\left(b-\frac{\eta }{\bar{\gamma }}\right)}^{c_{1}+k-i}\cdot {\left(b-\frac{\eta }{\bar{\gamma }}+\frac{\eta \beta_{0}}{\left(1+\beta_{0}\right)\bar{\gamma }}\right)}^{c_{2}-k-a+i} \end{array}$$ (24)

*where* *c*_1_ = *Lm* − *ζ*, *c*_2_ = −*Lm*, *and* (⋅)_*n*_ *is the pochhammer symbol*, *i.e.*, (*x*)_*n*_ = *x*(*x* − 1)(*x* − 2)⋯(*x* − *n* + 1).

*Proof*: See Appendix.

**Lemma 2** *Consider the sum of L independent non-identically distributed squared* *κ*-*μ* *shadowed variates with the instantaneous SINR* *γ*, *and the complementary IG-MGF of* *γ* *can be expressed as*

$$\begin{array}{ll} {\tilde{G}}_{\gamma }\left(a,b;\epsilon \right) & ={\left(-1\right)}^{a}C{\left(\frac{\eta }{\bar{\gamma }}\right)}^{\zeta }\frac{\epsilon^{a+\zeta }}{\Gamma (a+\zeta +1)}\sum_{k=0}^{\infty }{\bar{D}}_{k}\\ & \cdot \sum_{i=0}^{a}\frac{a!{\left(c_{1}+k-i+1\right)}_{i}\;{\left(c_{2}-k-a+i+1\right)}_{a-i}}{i!(a-i)!}\\ & \cdot \Phi_{2}^{\left(2\right)}\left[-c_{1}-k+i,-c_{2}+k+a-i;a+\zeta +1;\left(b-\frac{\eta }{\bar{\gamma }}\right)\epsilon ,\right.\\ & \left.\left(b-\frac{\eta }{\bar{\gamma }}+\frac{\eta \beta_{0}}{\left(1+\beta_{0}\right)\bar{\gamma }}\right)\epsilon \right] \end{array}$$ (25)

*where* $\Phi_{2}^{\left(2\right)}\left(\cdot \right)$ *is the multivariate confluent hypergeometric function* [32].

*Proof*: See Appendix.

**Corollary 1** *Under the conditions of Lemma 1-2*, *the IG-MGF of the sum of L independent but arbitrarily distributed squared* *κ*-*μ* *shadowed variates is derived by*

$$G_{\gamma }\left(a,b;\epsilon \right)=G_{\gamma }\left(a,b;0\right)-{\tilde{G}}_{\gamma }\left(a,b;\epsilon \right)$$ (26)

*where* $G_{\gamma }\left(a,b;0\right)$ *and* ${\tilde{G}}_{\gamma }\left(a,b;\epsilon \right)$ *are respectively given in* Eqs (24) *and* (25).

### IG-MGF of the correlated non-identically distributed *κ*-*μ* shadowed variates

Assume that the dominant components of the *κ*-*μ* shadowed variates are correlated, and let *ρ*_*i*,*j*_ denote the correlation coefficients between ${\bar{\xi }}_{i}^{2}$ and ${\bar{\xi }}_{j}^{2}$, i.e.,

$$\rho_{ij}=\rho_{ji}=\frac{Cov({\bar{\xi }}_{i}^{2},{\bar{\xi }}_{j}^{2})}{\sqrt{Var\left({\bar{\xi }}_{i}^{2}\right)Var\left({\bar{\xi }}_{j}^{2}\right)}}\;i,j=1,2,\ldots$$ (27)

The PDF of $Z=\sum_{l=1}^{L}{\bar{\xi }}_{l}^{2}$ is given by [28]

$$f_{Z}\left(z\right)=\prod_{l=1}^{L}{\left(\frac{\lambda_{0}}{\lambda_{l}}\right)}^{m}\sum_{k=0}^{\infty }\frac{\delta_{k}z^{mL+k-1}e^{-z/\lambda_{0}}}{\lambda_{0}^{mL+k}\Gamma \left(mL+k\right)}$$ (28)

where the coefficients *δ*_*k*_ can be recursively derived by

$$\delta_{k+1}=\frac{m}{k+1}\sum_{i=1}^{k+1}\left[\sum_{j=1}^{L}{\left(1-\frac{\lambda_{0}}{\lambda_{j}}\right)}^{i}\right]\delta_{k+1-i},\;k=0,1,2,\ldots$$ (29)

where *δ*_0_ = 1. *λ*_*l*_, *l* = 1, 2, …, *L*, are the eigenvalues of the matrix *DB*, and *λ*_0_ is the smallest one of ${\left\{\lambda_{l}\right\}}_{l=1}^{L}$. *D* is a diagonal matrix whose entries are *μ*_*l*_*κ*_*l*_/*m*, and *B* is a positive definite matrix given by

$$B=\left[\begin{array}{cccc} 1 & \sqrt{\rho_{12}} & \cdots & \sqrt{\rho_{1L}}\\ \sqrt{\rho_{21}} & 1 & \cdots & \sqrt{\rho_{2L}}\\ \vdots & \vdots & \vdots & \vdots \\ \sqrt{\rho_{L1}} & \sqrt{\rho_{L2}} & \cdots & 1 \end{array}\right]$$ (30)

As shown in [28] and [33], the PDF of Eq (28) is a converging power series. In the Rician shadowed fading channels in [34], a similar model is also investigated for the scenario of the correlated shadowing components.

Averaged upon *Z*, the unconditional PDF of *γ* can be obtained by conditional PDF in Eq (15) as follows

$$f_{\gamma }\left(\gamma \right)=\bar{C}{\left(\frac{\eta }{\bar{\gamma }}\right)}^{\zeta }\gamma^{\zeta -1}e^{-\frac{\eta }{\bar{\gamma }}\gamma }\sum_{k=0}^{\infty }{\tilde{D}}_{k}^{{}^{\prime }}\cdot {}_{1}F_{1}\left(mL+k;\zeta ;\frac{\eta \lambda_{0}\gamma }{\left(1+\lambda_{0}\right)\bar{\gamma }}\right)$$ (31)

where $\bar{C}=\prod_{l=1}^{L}{\left(\lambda_{0}/\lambda_{l}\right)}^{m}$, ${\tilde{D}}_{k}^{{}^{\prime }}=\delta_{k}/{\left(1+\lambda_{0}\right)}^{mL+k}\Gamma \left(\zeta \right)$. Based on the definition of MGF and the properties of the Laplace transform, it follows that

$$\begin{array}{ll} M_{\gamma }(s) & =ℒ[f_{\gamma }(\gamma );-s]\\ & =\bar{C}{\left(\frac{\eta }{\bar{\gamma }}\right)}^{\zeta }\sum_{k=0}^{\infty }{\tilde{D}}_{k}^{\prime }ℒ\left[\gamma^{\zeta -1}{}_{1}F_{1}\left(mL+k;\zeta ;\frac{\eta \lambda_{0}\gamma }{(1+\lambda_{0})\bar{\gamma }}\right);\frac{\eta }{\bar{\gamma }}-s\right]\\ & =\bar{C}{\left(\frac{\eta }{\bar{\gamma }}\right)}^{\zeta }\sum_{k=0}^{\infty }{\bar{D}}_{k}^{\prime }{\left(\frac{\eta }{\bar{\gamma }}-s\right)}^{mL+k-\zeta }{\left(\frac{\eta }{\bar{\gamma }}-\frac{\eta \lambda_{0}}{(1+\lambda_{0})\bar{\gamma }}-s\right)}^{-(mL+k)} \end{array}$$ (32)

where ${\bar{D}}_{k}^{{}^{\prime }}=\delta_{k}/{\left(1+\lambda_{0}\right)}^{mL+k}$. We can use another method to calculate the diversity order, assuming that $\bar{\gamma }$ is very large, so $\eta /\bar{\gamma }$ is very small, and the asymptotic value of the MGF is written as

$$M_{\gamma }\left(s\right)={\left(-1\right)}^{\zeta }C{\left(\frac{\eta }{\bar{\gamma }}\right)}^{\zeta }\sum_{k=0}^{\infty }{\bar{D}}_{k}s^{-\zeta }$$ (33)

**Fig 1:**
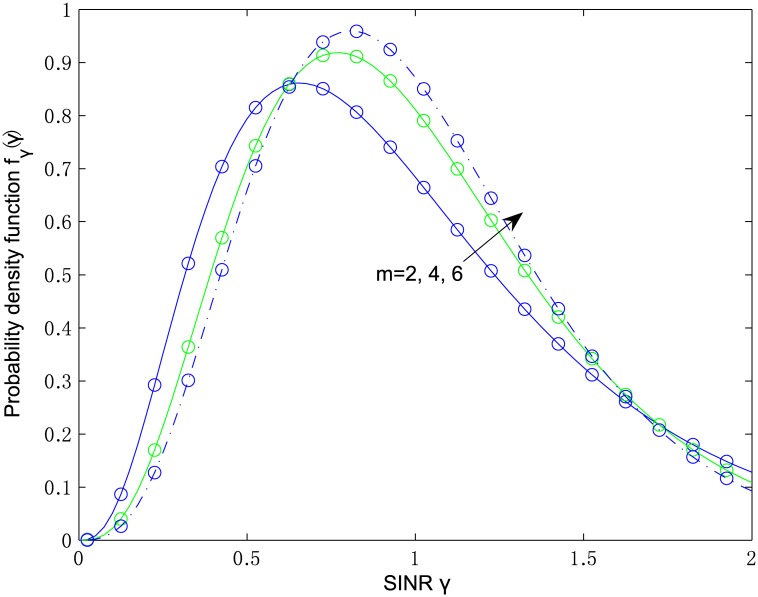
Analytical (-) and simulated (∘) probability density functions for *L* = 2, *κ*_*l*_ = 2, *μ*_*l*_ = 2, *m* = 2, 4, 6, and *ρ*_*ij*_ = 0.7^|*i*−*j*|^.

From Eq (33), it can be seen that the diversity order of the MRC scheme is $\zeta =\sum_{l=1}^{L}\mu_{l}$. The analytical values of the PDF in Eq (31) and the simulated PDF are both plotted in Fig 1 versus the instantaneous SINR *γ* with parameters *κ*_*l*_ = 2, *μ*_*l*_ = 2, *m* = 2, 4, 6, *L* = 2, and correlation coefficients *ρ*_*ij*_ = 0.7^|*i*−*j*|^. It can be seen that the simulated PDF is in remarkable good agreement with the analytical PDF, which justifies the correctness of the deduced PDF results.

**Lemma 3** *Consider the sum of L correlated non-identically distributed squared* *κ*-*μ* *shadowed variates with the instantaneous SINR* *γ*. *For* *a* ≥ 0, *Re*(*b*) < 0, *the generalized MGF of* *γ*, *i.e.*, $G_{\gamma }\left(a,b;0\right)$, *can be expressed as*

$$\begin{array}{ll} G_{\gamma }\left(a,b;0\right) & ={\left(-1\right)}^{a}\bar{C}{\left(\frac{\eta }{\bar{\gamma }}\right)}^{\zeta }\sum_{k=0}^{\infty }{\bar{D}}_{k}^{{}^{\prime }}\sum_{i=0}^{a}\frac{a!{\left(c_{1}+k-i+1\right)}_{i}\;{\left(c_{2}-k-a+i+1\right)}_{a-i}}{i!(a-i)!}\\ & \cdot {\left(-b+\frac{\eta }{\bar{\gamma }}\right)}^{c_{1}+k-i}\cdot {\left(-b+\frac{\eta }{\bar{\gamma }}-\frac{\eta \lambda_{0}}{\left(1+\lambda_{0}\right)\bar{\gamma }}\right)}^{c_{2}-k-a+i} \end{array}$$ (34)

*where* *c*_1_, *c*_2_ *take the same values as in Lemma 1*.

**Lemma 4** *Consider the sum of L correlated non-identically distributed squared* *κ*-*μ* *shadowed variates with the instantaneous SINR* *γ*, *and the complementary IG-MGF of* *γ* *is given by*

$$\begin{array}{ll} {\tilde{G}}_{\gamma }\left(a,b;\epsilon \right) & ={\left(-1\right)}^{a}\bar{C}{\left(\frac{\eta }{\bar{\gamma }}\right)}^{\zeta }\frac{\epsilon^{a+\zeta }}{\Gamma (a+\zeta +1)}\sum_{k=0}^{\infty }{\bar{D}}_{k}^{{}^{\prime }}\\ & \cdot \sum_{i=0}^{a}\frac{a!{\left(c_{1}+k-i+1\right)}_{i}\;{\left(c_{2}-k-a+i+1\right)}_{a-i}}{i!(a-i)!}\\ & \cdot \Phi_{2}^{\left(2\right)}\left[-c_{1}-k+i,-c_{2}+k+a-i;a+\zeta +1;\left(b-\frac{\eta }{\bar{\gamma }}\right)\epsilon ,\right.\\ & \left.\left(b-\frac{\eta }{\bar{\gamma }}+\frac{\eta \lambda_{0}}{\left(1+\lambda_{0}\right)\bar{\gamma }}\right)\epsilon \right] \end{array}$$ (35)

*Proof*: The proof of Lemma 3-4 follows along the same lines as the proof of Lemma 1-2, and here it is omitted for brevity.

Following the same procedure as the independent case, we can obtain the IG-MGF $G_{\gamma }\left(a,b;\epsilon \right)$ of the sum of *L* correlated non-identically distributed squared *κ*-*μ* shadowed variates from the analysis of generalized MGF $G_{\gamma }\left(a,b;0\right)$ and complementary IG-MGF ${\tilde{G}}_{\gamma }\left(a,b;\epsilon \right)$, i.e.,

$$G_{\gamma }\left(a,b;\epsilon \right)=G_{\gamma }\left(a,b;0\right)-{\tilde{G}}_{\gamma }\left(a,b;\epsilon \right)$$ (36)

where $G_{\gamma }\left(a,b;0\right)$ and ${\tilde{G}}_{\gamma }\left(a,b;\epsilon \right)$ are given in Eqs (34) and (35).

## Outage Probability Calculation

**Fig 2:**
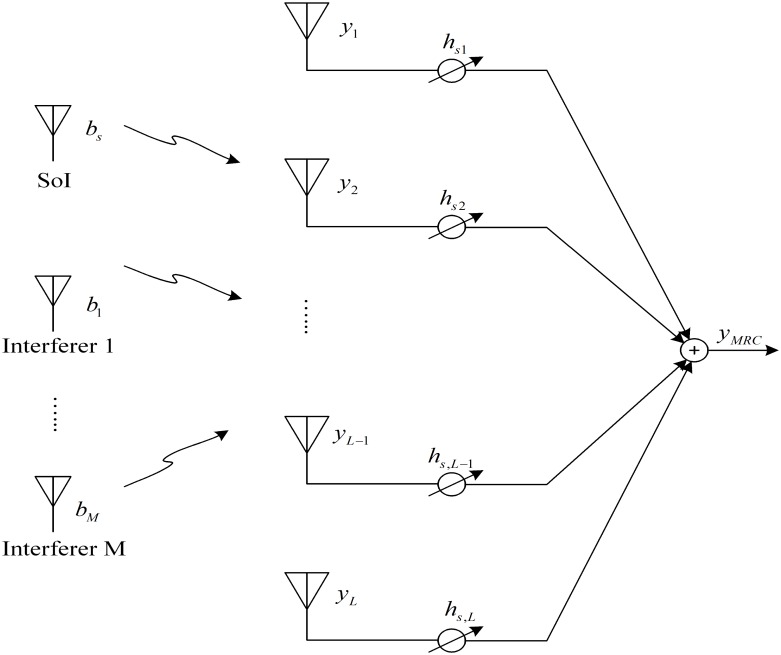
System model.

As shown in Fig 2, consider a wireless communication system with *L* receive antennas where the received desired signal at every antenna is assumed to be corrupted by *M* CCI terms and zero-mean additive white Gaussian noise (AWGN) with variance $\sigma_{0}^{2}$. The received desired signal at each antenna is assumed to experience *κ*-*μ* shadowed fading, while interfering signals are subject to identically distributed Rayleigh fading, and every interferer is assumed to have arbitrary power. Denote *h*_*s*_ = [*h*_*s*1_ *h*_*s*2_ … *h*_*sL*_]^*T*^ and *h*_*i*_ = [*h*_*i*1_ *h*_*i*2_ … *h*_*iL*_]^*T*^ as the channel gains of the desired and the *i*th interfering signal at the receive antennas, respectively, where *T* represents vector transposition. The received *L*-element baseband signal *y* can be expressed as

$$y=h_{s}b_{s}+\sum_{i=1}^{M}\sqrt{P_{i}}h_{i}b_{i}+n$$ (37)

where *b*_*i*_ and *b*_*s*_ represent the transmitted symbols from the *i*th interfering and desired user, respectively. *P*_*i*_ denotes the average power of the *i*th interfering signal at each antenna, and *n* is the *L*-dimensional noise vector. For simplicity, it is considered that ‖*b*_*s*_‖ = 1, ‖*b*_*i*_‖ = 1, *i* = 1, 2, …, *L*.

For MRC scheme, the output signal can be obtained by multiplying the channel gains associated with the desired user, i.e.,

$$y_{MRC}=h_{s}^{H}y={∥h_{s}∥}^{2}b_{s}+\sum_{i=1}^{M}\sqrt{P_{i}}h_{s}^{H}h_{i}b_{i}+h_{s}^{H}n$$ (38)

where the superscript *H* refers to Hermitian transposition. Thus, the received SINR with MRC will be

$$\gamma =\frac{∥h_{s}{∥}^{2}}{\sum_{i=1}^{M}P_{i}{\left|v_{i}\right|}^{2}+\sigma_{0}^{2}}=\frac{W}{Y+\sigma_{0}^{2}}$$ (39)

where $v_{i}=h_{s}^{H}h_{i}/∥h_{s}∥$, $W=\sum_{i=1}^{L}{\left|h_{si}\right|}^{2}$, and $Y=\sum_{i=1}^{M}P_{i}{\left|v_{i}\right|}^{2}$. Therefore, *W* is distributed as the sum of *L* squared *κ*-*μ* shadowed random variables, and *Y* is the total instantaneous power of the interfering signals.

Divide the total interferers into *K* groups, and assume there are *n*_*i*_ interferers in the *i*th group with the same mean power *P*_*i*_. The corresponding outage probability can be computed as follows [25]

$$\begin{array}{ll} P_{out} & =Pr\left\{\frac{W}{Y+\sigma_{0}^{2}}<{\bar{\gamma }}_{0}\right\}=Pr\left\{\frac{\gamma }{Y+\sigma_{0}^{2}}<\gamma_{0}\right\}=\int_{0}^{\gamma_{0}\sigma_{0}^{2}}f_{\gamma }\left(\gamma \right)d\gamma \\ & +\sum_{i=1}^{K}\sum_{j=1}^{n_{i}}\sum_{k=0}^{n_{i}-j}\sum_{l=0}^{k}E_{ij}\frac{e^{\sigma_{0}^{2}/P_{i}}{\left(-\sigma_{0}^{2}\right)}^{k-l}}{l!\left(k-l\right)!P_{i}^{k}\gamma_{0}^{l}}\int_{\gamma_{0}\sigma_{0}^{2}}^{\infty }\gamma^{l}e^{-\frac{\gamma }{\gamma_{0}P_{i}}}f_{\gamma }\left(\gamma \right)d\gamma \end{array}$$ (40)

where *γ*_0_ is the threshold level, and $\gamma_{0}=\bar{\gamma }{\bar{\gamma }}_{0}/E\left[W\right]$. *E*_*ij*_ are derived by

$$E_{ij}={\left(-1\right)}^{j-1}\sum_{\Omega_{A}}\prod_{k=1,k\neq i}^{K}\left[\begin{array}{c} n_{k}+q_{k}-1\\ n_{k}-1 \end{array}\right]\frac{P_{k}^{q_{k}}P_{i}^{n_{k}}}{{\left(P_{i}-P_{k}\right)}^{n_{k}+q_{k}}}$$ (41)

where Ω_*A*_ represents the set satisfying $\Omega_{A}=\{\left(q_{1},q_{2},\ldots ,q_{K}\right):q_{k}\in N\cup \left\{0\right\},q_{i}=0,\sum_{k=1}^{K}q_{k}=j-1$}, and N is the set of positive integers.

The outage probability in Eq (40) is given by the sum of two incomplete integrals. The first one denotes the outage probability without the interfering signals, which can be expressed by the CDF of *γ*, i.e., ${\tilde{G}}_{\gamma }\left(0,0;\gamma_{0}\sigma_{0}^{2}\right)$. The second one represents outage probability under the presence of interference, which can be derived by the incomplete generalized MGF of *γ*, *G*_*γ*_(⋅, ⋅; ⋅). Obviously, the integrals equals the IG-MGF of *γ*, i.e.,

$$\int_{\gamma_{0}\sigma_{0}^{2}}^{\infty }\gamma^{l}e^{-\frac{\gamma }{\gamma_{0}P_{i}}}f_{\gamma }\left(\gamma \right)d\gamma =G_{\gamma }\left(l,-\frac{1}{\gamma_{0}P_{i}};\gamma_{0}\sigma_{0}^{2}\right)$$ (42)

Therefore, by substituting Eq (42) into Eq (40), the final outage probability of MRC for *κ*-*μ* shadowed fading channels with Rayleigh-faded interferers is obtained as follows

$$P_{out}={\tilde{G}}_{\gamma }\left(0,0;\gamma_{0}\sigma_{0}^{2}\right)+\sum_{i=1}^{K}\sum_{j=1}^{n_{i}}\sum_{k=0}^{n_{i}-j}\sum_{l=0}^{k}E_{ij}\frac{e^{\sigma_{0}^{2}/P_{i}}{\left(-\sigma_{0}^{2}\right)}^{k-l}}{l!\left(k-l\right)!P_{i}^{k}\gamma_{0}^{l}}G_{\gamma }\left(l,-\frac{1}{\gamma_{0}P_{i}};\gamma_{0}\sigma_{0}^{2}\right)$$ (43)

where the coefficients *E*_*ij*_ are computed by Eq (41). When *W* is distributed as the sum of *L* independent but arbitrarily distributed squared *κ*-*μ* shadowed variates, the outage probability *P*_*out*_ can be obtained by Lemma 1-2 and Corollary 1, and for the correlated case, *P*_*out*_ is given by Lemma 3-4. Obviously, the outage probability in Eq (43) is essentially expressed by the multivariate confluent hypergeometric function.

Note that to compute the outage probability, the incomplete generalized MGF of *γ*, i.e., *G*_*γ*_(⋅, ⋅; ⋅), is essential, so it is necessary to calculate the multivariate confluent hypergeometric function $\Phi_{2}^{\left(2\right)}\left(\cdot \right)$. However, $\Phi_{2}^{\left(2\right)}\left(\cdot \right)$ is not implemented in Matlab, and it can be evaluated by the numerical techniques, i.e., the inverse Laplace transform. For the independent case, in the proof of Lemma 2, we obtain that

$$\begin{array}{l} L^{-1}\left[\frac{1}{s}{\left(s-b+\frac{\eta }{\bar{\gamma }}\right)}^{c_{1}+k-i}{\left(s-b+\frac{\eta }{\bar{\gamma }}-\frac{\eta \lambda_{0}}{\left(1+\lambda_{0}\right)\bar{\gamma }}\right)}^{c_{2}-k-a+i}\right]\\ =\frac{\epsilon^{a+\zeta }}{\Gamma (a+\zeta +1)}\Phi_{2}^{\left(2\right)}\left[-c_{1}-k+i,-c_{2}+k+a-i;a+\zeta +1;\left(b-\frac{\eta }{\bar{\gamma }}\right)\epsilon ,\right.\;\;\;\left.\left(b-\frac{\eta }{\bar{\gamma }}+\frac{\eta \beta_{0}}{\left(1+\beta_{0}\right)\bar{\gamma }}\right)\epsilon \right] \end{array}$$ (44)

Using the numerical technique in [1] yields

$$\begin{array}{l} \frac{\epsilon^{a+\zeta }}{\Gamma (a+\zeta +1)}\Phi_{2}^{\left(2\right)}[-c_{1}-k+i,-c_{2}+k+a-i;a+\zeta +1;\left(b-\frac{\eta }{\bar{\gamma }}\right)\epsilon ,\\ \left(b-\frac{\eta }{\bar{\gamma }}+\frac{\eta \beta_{0}}{(1+\beta_{0})\bar{\gamma }}\right)\epsilon ]\\ =\frac{2^{-U}e^{A/2}}{\epsilon }\sum_{u=0}^{U}\left[\begin{array}{c} U\\ u \end{array}\right]\sum_{v=0}^{V+u}\frac{{\left(-1\right)}^{v}}{\alpha_{v}}Re\{\frac{1}{\frac{A+2\pi jv}{2\epsilon }}{\left(\frac{A+2\pi jv}{2\epsilon }-b+\frac{\eta }{\bar{\gamma }}\right)}^{c_{1}+k-i}\\ {\left(\frac{A+2\pi jv}{2\epsilon }-b+\frac{\eta }{\bar{\gamma }}-\frac{\eta \beta_{0}}{(1+\beta_{0})\bar{\gamma }}\right)}^{c_{2}-k-a+i}\}+E(A,U,V) \end{array}$$ (45)

where *E*(*A*, *U*, *V*) is the error term. For *v* = 0, *α*_*n*_ = 2, when *v* = 1, 2, …, *V*, *α*_*n*_ = 1. Obviously, once $\Phi_{2}^{\left(2\right)}\left(\cdot \right)$ is obtained, the incomplete generalized MGF ${\tilde{G}}_{\gamma }\left(a,b;\epsilon \right)$ can be easily evaluated.

## Simulation Results

In this section, analytical derivations of the outage probability are validated against the simulation results. In the physical model of the *κ* − *μ* shadowed distribution, *μ* is a natural number, so simulations are carried out with natural values of *μ*. However, for real number case, the pdf or cdf based method can be used for simulations. In fact, all the analytical results derived in the paper are valid for arbitrary real number *μ*. The outage probability is shown versus the average SINR normalized by *γ*_0_, i.e.,

$$\frac{\bar{\gamma }}{\gamma_{0}}\left[dB\right]=10\log_{10}\left(\frac{W_{s}}{\gamma_{0}\left(\sum P_{i}+\sigma_{0}^{2}\right)}\right)$$

for different values of shaping parameter *m*, and several values of *ρ* and *L*. In the particular example, the power of three interferers are chosen as *P*_1_ = 1/6, *P*_2_ = 1/8, *P*_3_ = 1/9, while the power of background noise is 1/10.

**Fig 3:**
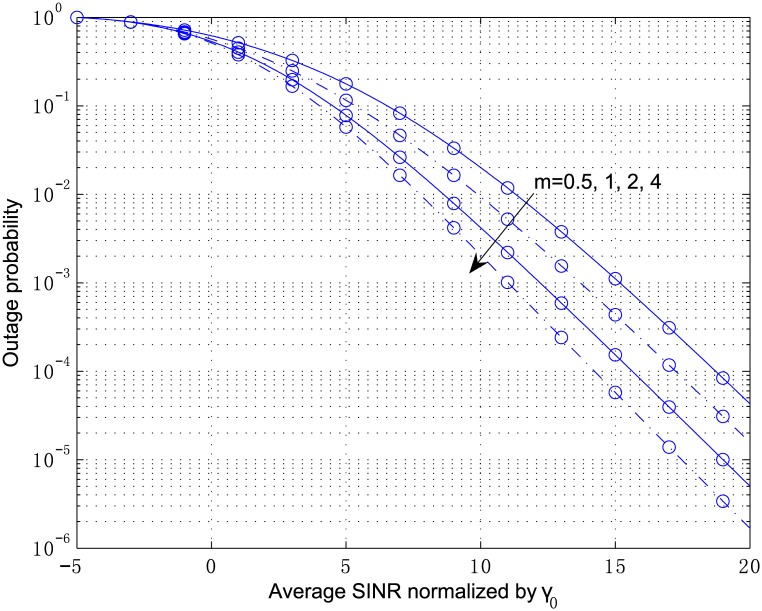
Outage probability versus average SINR normalized by *γ*_0_ for MRC with *L* = 2, *κ*_1_ = 2.1, *κ*_2_ = 3.6, *μ*_1_ = 2, and *μ*_2_ = 1.

**Fig 4:**
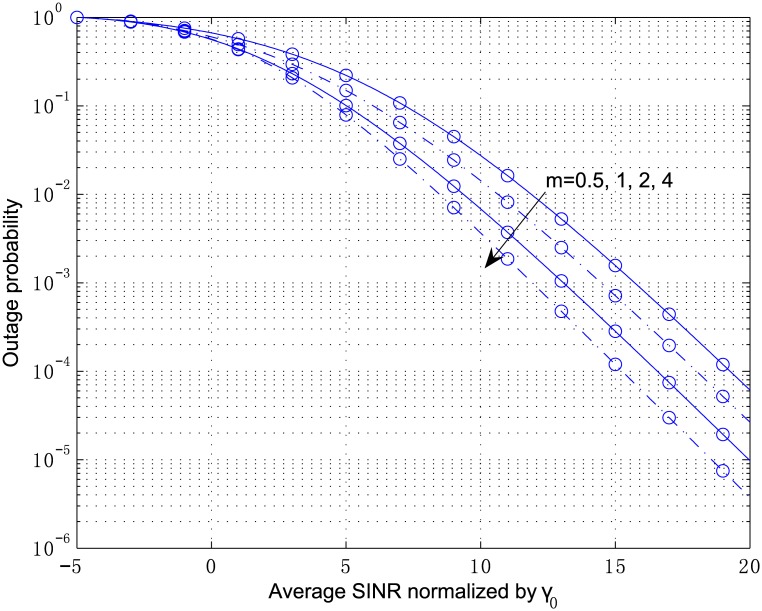
Outage probability versus average SINR normalized by *γ*_0_ for MRC with *L* = 2, *κ*_1_ = 2.1, *κ*_2_ = 3.6, *μ*_1_ = 2, *μ*_2_ = 1, and *ρ*_12_ = 0.8.

Fig 3 plots the outage probability of a 2-antenna MRC receiver from Eqs (24)–(26) and (43) for the independent but arbitrarily distributed case, where the parameters are chosen as *κ*_1_ = 2.1, *κ*_2_ = 3.6, *μ*_1_ = 2, and *μ*_2_ = 1. The multivariate confluent hypergeometric function $\Phi_{2}^{\left(2\right)}$ can be efficiently computed by the inverse Laplace transform in Eq (45), where the parameters are chosen as *A* = 10ln10, *U* = 11, and *V* = 20. The outage probability in Fig 4 corresponds to the correlated fading computed by Eqs (34)–(36) and (43) with the correlation coefficient *ρ*_12_ = 0.8. In both figures, the analytical results are in perfect agreement with the simulation results. In addition, correlation among the diversity branches yields a non-negligible degradation of the outage performance. The results for the scenario show that the shadowing has a significant influence on the system performance although the LOS strength at each branch, i.e., *κ*, is below 5 *dB*.

**Fig 5:**
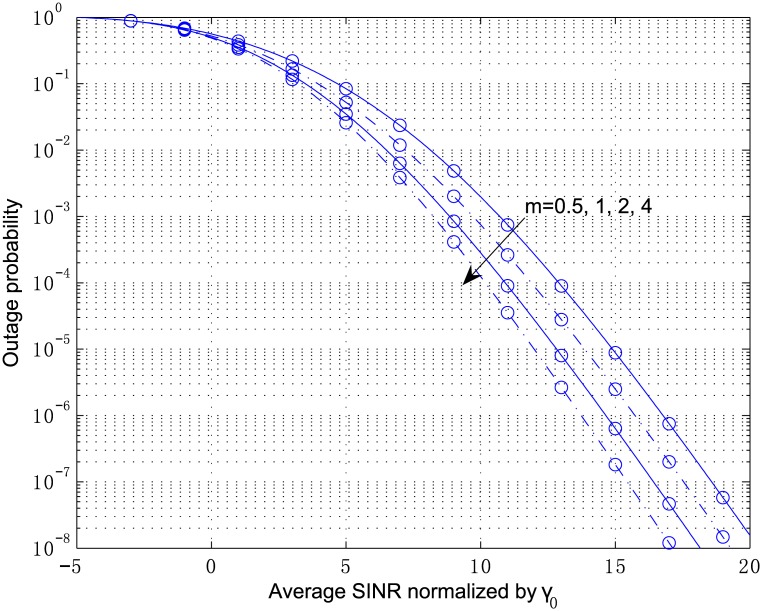
Outage probability versus average SINR normalized by *γ*_0_ for MRC with *L* = 3, *κ*_1_ = 2.1, *κ*_2_ = 3.6, *κ*_3_ = 1.2, *μ*_1_ = 2, *μ*_2_ = 1, and *μ*_3_ = 3.

**Fig 6:**
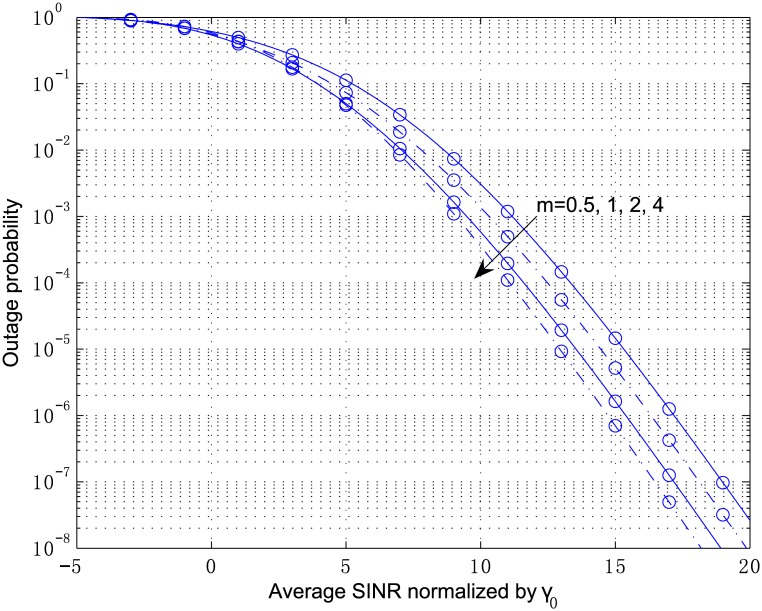
Outage probability versus average SINR normalized by *γ*_0_ for MRC with *L* = 3, *κ*_1_ = 2.1, *κ*_2_ = 3.6, *κ*_3_ = 1.2, *μ*_1_ = 2, *μ*_2_ = 1, *μ*_3_ = 3, *ρ*_12_ = 0.6, *ρ*_13_ = 0.5, and *ρ*_23_ = 0.4.

In Fig 5, a triple-branch MRC scenario is considered, in which the parameters are considered with *κ*_1_ = 2.1, *κ*_2_ = 3.6, *κ*_3_ = 1.2, *μ*_1_ = 2, *μ*_2_ = 1, *μ*_3_ = 3. In this figure, the analytical and simulation OP results are plotted versus the average SNIR per branch for the independent MRC. The outage probability corresponding to the correlated fading is plotted in Fig 6 with correlation parameters *ρ*_12_ = 0.6, *ρ*_13_ = 0.5, *ρ*_23_ = 0.4. It can be seen that the simulated outage probability matches the analytical results at all SINR values in these figures. Moreover, the antenna correlation has a great influence on the outage probability of the receiver. However, for the MRC scheme, the diversity order is independent of the antenna correlation. As in the 2-antenna case, the system performance is adversely affected by the shadowing parameter *m*.

**Fig 7:**
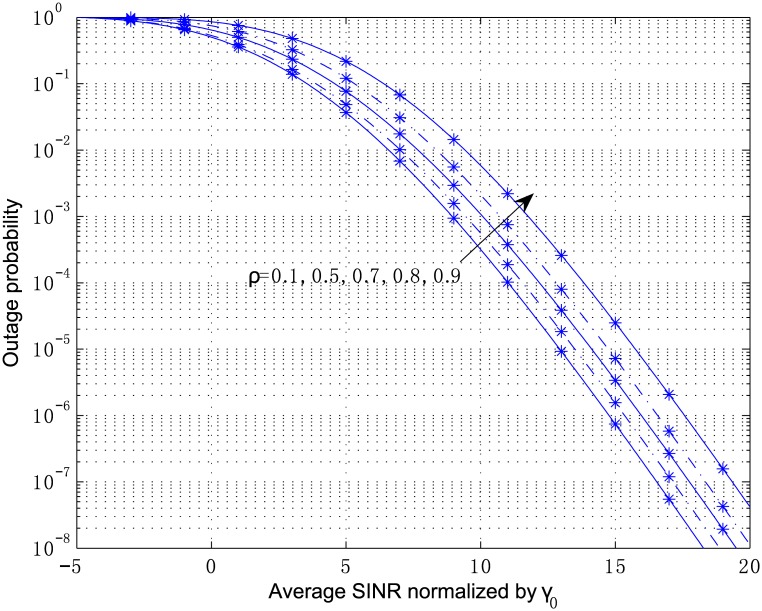
Outage probability versus average SINR normalized by *γ*_0_ for MRC with *L* = 3, *κ*_1_ = 2.1, *κ*_2_ = 3.6, *κ*_3_ = 1.2, *μ*_1_ = 2, *μ*_2_ = 1, and *μ*_3_ = 3, *ρ*_*ij*_ = *ρ*^|*i*−*j*|^, and *ρ* = 0.1, 0.5, 0.7, 0.8, 0.9.

**Fig 8:**
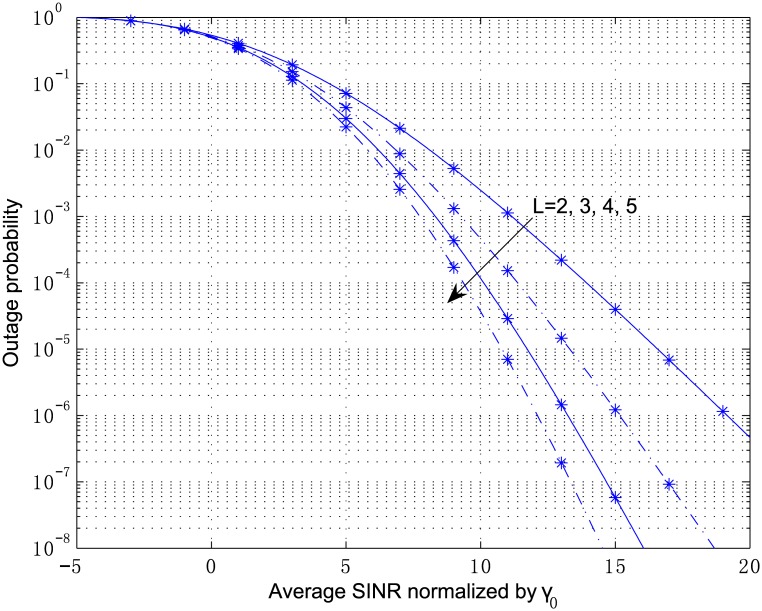
Outage probability versus average SINR normalized by *γ*_0_ for MRC with *κ*_*l*_ = 5, *μ*_*l*_, *m* = 2, *ρ*_*ij*_ = 0.5^|*i*−*j*|^, *L* = 2, 3, 4, 5.

Fig 7 shows the effect of correlation parameter on the outage probability of the MRC receiver, where *κ*_1_ = 2.1, *κ*_2_ = 3.6, *κ*_3_ = 1.2, *μ*_1_ = 2, *μ*_2_ = 1, and *μ*_3_ = 3, *ρ*_*ij*_ = *ρ*^|*i*−*j*|^, *ρ* = 0.1, 0.5, 0.7, 0.8, 0.9, *L* = 3. It can be seen that the receiver performance degrades with the increase of the correlation coefficient. The analytical outage probability for *κ*_*l*_ = 5, *μ*_*l*_ = 2, *m* = 2, *ρ*_*ij*_ = 0.5^|*i*−*j*|^, *L* = 2, 3, 4, 5 is plotted in Fig 8, and the simulated OP is also shown in the figure versus normalized average SINR for the same parameters. It can be observed that the simulated OP is in a good coincidence with the analytical OP at all SINR values in the figure. Furthermore, for MRC scheme the results in Fig 8 show that the system performance improves with the increase of antenna number, which means the increasing slope of the curves, and otherwise diminishing appears as the antenna number decreases.

## Conclusions

This paper investigates the outage probability of the *κ* − *μ* shadowed fading model in presence of CCI and the background noise. First, it is assumed that the shadowing components of the *κ* − *μ* shadowed distribution are independent, and several statistical characterizations are derived for MRC, i.e., probability density function and incomplete generalized moment-generating functions. Based on the incomplete generalized MGF, closed-form expressions for the OP of MRC can be derived for this scenario. Further, the OP analysis can be generalized to the correlated case, where the shadowing components are assumed to be correlated with each other. It is shown that the OP results for MRC in *κ* − *μ* shadowed fading channels can be essentially expressed in terms of the multivariate confluent hypergeometric function. In the simulations, it can be computed by the numerical techniques, i.e., the inverse Laplace transform. Finally, the statistical results are validated against the simulations.

## Appendix

**Proof of Lemma 1**

The Laplace transform of *f*_*γ*_(*γ*) is defined as

$$L\left[f_{\gamma }\left(\gamma \right);s\right]=\int_{0}^{\infty }e^{-\gamma s}f_{\gamma }\left(\gamma \right)d\gamma =M_{\gamma }\left(-s\right)$$ (46)

The *a*th order derivative of *M*_*γ*_(−*s*) is

$$\frac{d^{a}}{ds^{a}}M_{\gamma }\left(-s\right)={\left(-1\right)}^{a}\int_{0}^{\infty }\gamma^{a}e^{-\gamma s}f_{\gamma }\left(\gamma \right)d\gamma$$ (47)

In view of Eqs (10) and (47), the generalized MGF $G_{\gamma }\left(a,b;0\right)$ can be derived by $G_{\gamma }\left(a,b;0\right)=L\left[\gamma^{a}f_{\gamma }\left(\gamma \right);s\right]{|}_{s=-b}$, i.e.,

$$G_{\gamma }\left(a,b;0\right)={\left(-1\right)}^{a}\frac{d^{a}}{ds^{a}}M_{\gamma }{\left(-s\right)|}_{s=-b}$$ (48)

Since

$$M_{\gamma }\left(-s\right)=C{\left(\frac{\eta }{\bar{\gamma }}\right)}^{\zeta }\sum_{k=0}^{\infty }{\bar{D}}_{k}M_{1}\left(-s\right)$$ (49)

where

$$M_{1}\left(-s\right)={\left(s+\frac{\eta }{\bar{\gamma }}\right)}^{c_{1}+k}{\left(s+\frac{\eta }{\bar{\gamma }}-\frac{\eta \beta_{0}}{\left(1+\beta_{0}\right)\bar{\gamma }}\right)}^{c_{2}-k}$$ (50)

It is true that

$$\begin{array}{ll} \frac{d^{a}}{ds^{a}}M_{1}\left(-s\right) & =\sum_{i=0}^{a}\frac{a!{\left(c_{1}+k-i+1\right)}_{i}\;{\left(c_{2}-k-a+i+1\right)}_{a-i}}{i!(a-i)!}{\left(s+\frac{\eta }{\bar{\gamma }}\right)}^{c_{1}+k-i}\\ & \cdot {\left(s+\frac{\eta }{\bar{\gamma }}-\frac{\eta \beta_{0}}{\left(1+\beta_{0}\right)\bar{\gamma }}\right)}^{c_{2}-k-a+i} \end{array}$$ (51)

From Eqs (48)–(51), we obtain

$$\begin{array}{ll} G_{\gamma }\left(a,b;0\right) & ={\left(-1\right)}^{a}C{\left(\frac{\eta }{\bar{\gamma }}\right)}^{\zeta }\sum_{k=0}^{\infty }{\bar{D}}_{k}\sum_{i=0}^{a}\frac{a!{\left(c_{1}+k-i+1\right)}_{i}\;{\left(c_{2}-k-a+i+1\right)}_{a-i}}{i!(a-i)!}\\ & {\left.{\left(s+\frac{\eta }{\bar{\gamma }}\right)}^{c_{1}+k-i}\cdot {\left(s+\frac{\eta }{\bar{\gamma }}-\frac{\eta \beta_{0}}{\left(1+\beta_{0}\right)\bar{\gamma }}\right)}^{c_{2}-k-a+i}\right|}_{s=-b} \end{array}$$ (52)

Since ${\left(-1\right)}^{c_{1}+c_{2}}\cdot {\left(-1\right)}^{a}={\left(-1\right)}^{\zeta }$, Eq (24) can be obtained.

**Proof of Lemma 2**

The Laplace transform of the complementary IG-MGF ${\tilde{G}}_{\gamma }\left(a,b;\epsilon \right)$ is

$$\begin{array}{ll} ℒ[{\tilde{G}}_{\gamma }(a,b;\epsilon );\epsilon ,s] & =\frac{1}{s}ℒ\left[\gamma^{a}e^{b\gamma }f_{\gamma }(\gamma );\gamma ,s\right]\\ & =\frac{1}{s}\int_{0}^{\infty }\gamma^{a}e^{(-s+b)\gamma }f_{\gamma }(\gamma )d\gamma \end{array}$$ (53)

Since

$$M_{\gamma }\left(-s+b\right)=\int_{0}^{\infty }e^{(-s+b)\gamma }f_{\gamma }\left(\gamma \right)d\gamma$$ (54)

Differentiating Eq (54), it is easy to obtain

$$\frac{d^{a}}{ds^{a}}M_{\gamma }\left(-s+b\right)={\left(-1\right)}^{a}\int_{0}^{\infty }\gamma^{a}e^{(-s+b)\gamma }f_{\gamma }\left(\gamma \right)d\gamma$$ (55)

So Laplace transform of the complementary IG-MGF ${\tilde{G}}_{\gamma }\left(a,b;\epsilon \right)$ can be derived by

$$\begin{array}{ll} ℒ[{\tilde{G}}_{\gamma }(a,b;\epsilon );\epsilon ,s] & ={\left(-1\right)}^{a}\frac{1}{s}\frac{d^{a}}{ds^{a}}M_{\gamma }(-s+b)\\ & ={\left(-1\right)}^{a}\frac{1}{s}\frac{d^{a}}{ds^{a}}ℒ[f_{\gamma }(\gamma );\gamma ,s-b] \end{array}$$ (56)

In view of Eq (21), it follows that

$$\begin{array}{ll} {\left(-1\right)}^{a}\frac{1}{s}\frac{d^{a}}{ds^{a}}M_{\gamma }\left(-s+b\right) & ={\left(-1\right)}^{a}C{\left(\frac{\eta }{\bar{\gamma }}\right)}^{\zeta }\sum_{k=0}^{\infty }{\bar{D}}_{k}\sum_{i=0}^{a}\frac{a!{\left(c_{1}+k-i+1\right)}_{i}\;{\left(c_{2}-k-a+i+1\right)}_{a-i}}{i!(a-i)!}\\ & \cdot \frac{1}{s}{\left(s-b+\frac{\eta }{\bar{\gamma }}\right)}^{c_{1}+k-i}{\left(s-b+\frac{\eta }{\bar{\gamma }}-\frac{\eta \beta_{0}}{\left(1+\beta_{0}\right)\bar{\gamma }}\right)}^{c_{2}-k-a+i} \end{array}$$ (57)

Using the equation [31], it yields

$$\begin{array}{l} L^{-1}\left[\frac{1}{s}{\left(s-b+\frac{\eta }{\bar{\gamma }}\right)}^{c_{1}+k-i}{\left(s-b+\frac{\eta }{\bar{\gamma }}-\frac{\eta \beta_{0}}{\left(1+\beta_{0}\right)\bar{\gamma }}\right)}^{c_{2}-k-a+i}\right]\\ =\frac{\epsilon^{a+\zeta }}{\Gamma (a+\zeta +1)}\Phi_{2}^{\left(2\right)}\left[-c_{1}-k+i,-c_{2}+k+a-i;a+\zeta +1;\left(b-\frac{\eta }{\bar{\gamma }}\right)\epsilon ,\right.\;\;\;\left.\left(b-\frac{\eta }{\bar{\gamma }}+\frac{\eta \beta_{0}}{\left(1+\beta_{0}\right)\bar{\gamma }}\right)\epsilon \right] \end{array}$$ (58)

Using Eqs (56)–(58), the resulting expression (25) can be obtained after simple algebraic manipulations.
